# Large language models for ophthalmic examination understanding: from information extraction to clinical decision support

**DOI:** 10.3389/fmed.2026.1937970

**Published:** 2026-09-11

**Authors:** Gangyi Wang, Xuanqiao Lin, Yizhou Yang

**Affiliations:** 1 Department of Ophthalmology, Taizhou First People’s Hospital, Taizhou, Zhejiang, China; 2 Department of Ophthalmology, Eye, Ear, Nose, and Throat Hospital of Fudan University, Shanghai, China

**Keywords:** clinical decision support, decision making, large language model, multimodal large language model, ophthalmic examination, ophthalmic imaging

## Abstract

Ophthalmology relies on heterogeneous examination outputs, including device-generated reports, structured measurements, fundus photographs, optical coherence tomography (OCT), visual field plots, corneal imaging, and multimodal clinical data. Publicly available large language models (LLMs) and multimodal large language models (MLLMs) can process these inputs without study-specific ophthalmic training, creating opportunities for information extraction, interpretation, and clinical decision support. This Mini Review synthesizes 37 peer-reviewed studies evaluating such models across ophthalmic examination types and task layers. The evidence shows a consistent task gradient. Structured extraction and constrained calculations are generally more reliable than open-ended image interpretation, multimodal diagnosis, or treatment planning. Performance improves when inputs are standardized, clinical context is provided, and prompts or decision rules are constrained, but remains sensitive to report layout, image preprocessing, prompt design, and model updates. Common limitations include retrospective or public datasets, small or selected cohorts, limited external validation, possible training-data contamination, inconsistent reporting of prompts and model access, and a focus on technical accuracy rather than clinical outcomes. Clinical translation therefore requires safeguards matched to task risk: structured outputs and deterministic checks for extraction, traceable evidence and cross-device validation for interpretation, and independent verification, deferral mechanisms, and clinician oversight for decision support. Publicly available LLMs and MLLMs are evolving from report-parsing tools toward broader ophthalmic clinical assistants, but their use should follow a task-layered validation framework in which verification and human oversight increase with the clinical consequences of error.

## 1 Introduction

Ophthalmology is a highly data-intensive and visually driven specialty. Clinical decisions routinely rely on heterogeneous examination outputs, including optical biometry reports, corneal topography and tomography maps, visual field printouts, optical coherence tomography (OCT), fundus photographs and other multimodal clinical data (1–3). These outputs vary substantially in structure, ranging from numerical tables and reliability indices to spatial maps, cross-sectional images, and free-text clinical context. Their interpretation therefore requires not only accurate data extraction, but also integration of visual patterns, quantitative measurements, and domain-specific clinical reasoning (4).

Conventional artificial intelligence systems in ophthalmology are generally developed for narrowly defined tasks using task-specific training datasets. Although such models may perform well within a particular disease or imaging modality, their development often requires extensive annotation, dedicated computational pipelines, and retraining when the clinical task, device, or data format changes (5, 6). Publicly available large language models (LLMs) and multimodal large language models (MLLMs) introduce a different paradigm (7). General-purpose systems such as ChatGPT, Gemini, Claude, and DeepSeek can process combinations of text, images, tables, and structured data without study-specific ophthalmic training, potentially allowing the same model family to support multiple stages of an ophthalmic workflow (8–10).

Recent studies have extended beyond text-based knowledge assessment toward direct interpretation of ophthalmic examination outputs (9, 11). Publicly available LLMs and MLLMs have been evaluated for extracting parameters from biometry and visual field reports (12, 13), interpreting corneal topography and tomography (14), analyzing fundus photographs and OCT images (10, 15), integrating multimodal retinal or glaucoma cases (16), reproducing ophthalmic calculations, and supporting treatment or surgical planning (17). These applications span a continuum from extraction, through interpretation, to clinical decision support. However, these task layers are fundamentally different. Structured numerical extraction and constrained calculations are not directly comparable with open-ended image interpretation, multimodal diagnosis, or treatment recommendation, and errors at each layer may carry different clinical consequences.

In this review, we use the term LLM-enabled ophthalmic test-report understanding as an umbrella concept for the interpretation of clinically meaningful ophthalmic examination information by publicly available, off-the-shelf LLMs or MLLMs. Relevant inputs may include complete device-generated reports, PDFs or screenshots, structured examination data, or the visual outputs of ophthalmic tests themselves, such as fundus photography, OCT, corneal imaging, visual field plots, and external ocular photographs. The defining feature is therefore not a single report format, but the use of a general-purpose publicly accessible model without study-specific ophthalmic training or fine-tuning. This review therefore summarizes the emerging peer-reviewed evidence, organizes studies by examination type, input modality, and task layer, and highlights recurring limitations related to model variability, reproducibility, external validation, and clinical generalizability. We further propose a task-layered framework in which extraction, interpretation, and decision support are treated as progressively higher-risk applications requiring increasingly stringent verification, transparency, and human oversight.

## 2 Search scope and evidence appraisal

Using a structured narrative approach, PubMed, Web of Science, and Scopus were searched on August 20, 2026, for studies published from January 1, 2023, through July 31, 2026. The search combined terms related to large language models with ophthalmology-related terms. Database-specific search fields and complete search strategies are provided in Supplementary Table S1. Eligible inputs included device-generated reports, PDF files, screenshots, scanned printouts, structured examination data, ophthalmic images, and multimodal combinations of images and clinical text. The review focused on publicly accessible, off-the-shelf LLMs or MLLMs evaluated without study-specific ophthalmic training or fine-tuning by the investigators. Eligible tasks included information extraction, interpretation, diagnosis, calculation, and clinical decision support. Studies were excluded when the principal reported performance depended on an additional investigator-developed, trained, fine-tuned, or external AI component such that performance could not be attributed to the off-the-shelf LLM or MLLM itself. Studies primarily focused on ophthalmology-specific models developed, trained, or fine-tuned by the investigators were also excluded, as were reviews, editorials, conference abstracts without full reports, and preprints. In otherwise eligible studies containing specialty-trained or investigator-fine-tuned comparator models, only results from eligible off-the-shelf models were considered in the evidence synthesis.

**Figure 1:**
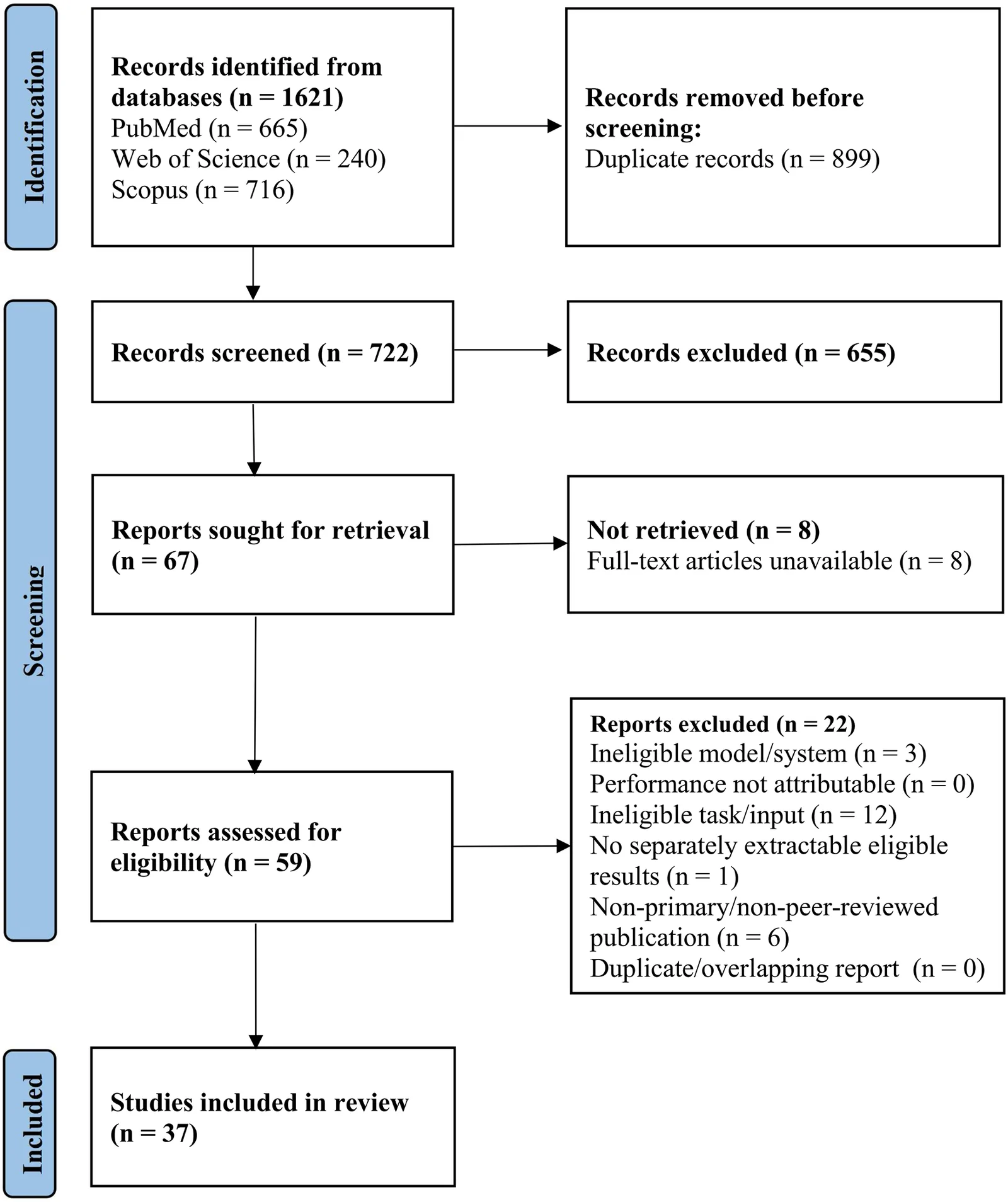
Study-selection flow diagram. Flow diagram showing study identification, screening, full-text eligibility assessment, and final inclusion.

Duplicate records were removed before screening. Records were screened by title and abstract, and potentially eligible reports subsequently underwent full-text assessment against the predefined inclusion and exclusion criteria. The complete study-selection process is shown in Figure 1.

A single conventional risk-of-bias tool was not applied because the evidence base included heterogeneous study types, including retrospective clinical cohorts, public image-dataset benchmarks, educational case repositories, examination-question datasets, and clinical decision-support studies. Instead, studies were appraised qualitatively across consistent domains: study design and sample size; source and representativeness of the input data; model reporting; prompt transparency and reproducibility; clarity of the reference standard; appropriateness of evaluation metrics; comparator use; external validation; and relevance to real-world clinical practice.

## 3 Evidence by task layer and report type

The searches identified 1,621 records (PubMed, *n* = 665; Web of Science, *n* = 240; Scopus, *n* = 716). After removal of 899 duplicate records, 722 records underwent title and abstract screening, of which 655 were excluded. Sixty-seven reports were sought for retrieval; 8 could not be retrieved, leaving 59 reports for full-text eligibility assessment. Twenty-two reports were excluded because of an ineligible model or system (*n* = 3), an ineligible task or input (*n* = 12), no separately extractable eligible results (*n* = 1), or a non-primary or non-peer-reviewed publication (*n* = 6). Thirty-seven studies were included in the final evidence synthesis (Figure 1).

The included studies (Supplementary Table S2) can be grouped into three task layers: information extraction, interpretation and diagnosis, and clinical decision support. Extraction tasks focus on identifying and structuring measurements from device-generated outputs. Interpretation tasks require recognition of clinically relevant findings from reports, images, or multimodal case data. Decision-support tasks extend to calculations, treatment recommendations, surgical planning, and other management decisions. The input formats also vary, including optical biometry reports, corneal tomography, visual field outputs, fundus photographs, OCT, external ocular photographs, structured clinical data, and image-text combinations (18). Because task complexity and input structure differ substantially, the evidence is summarized by task layer and report or examination type rather than by model alone.

### 3.1 Optical biometry and IOL planning

Optical biometry has been one of the earliest areas in which general-purpose LLMs were tested for ophthalmic report understanding. Tan et al. evaluated ChatGPT on 40 optical biometry reports and showed high extraction accuracy, although performance improved when the region of interest was explicitly specified, indicating sensitivity to report layout and image framing (12). Lin et al. extended this approach to toric IOL planning in 54 eyes. ChatGPT-5 Thinking showed the highest overall agreement for biometric parameters, including more reliable axis-related extraction, and generated the most consistent toric planning results (19).

Beyond report extraction, LLMs have also been tested for calculation and surgical decision support. Jun et al. evaluated ChatGPT and Gemini using structured biometric data from 180 eyes to reproduce SRK/T-based IOL power calculations. ChatGPT achieved a mean absolute error of 0.30 D, with 78.8% and 97.7% of predictions within ±0.50 D and ±1.00 D, respectively. This task, however, assessed formula replication rather than postoperative refractive prediction (20). Liu et al. evaluated ChatGPT using multimodal preoperative data from 146 eyes for IOL type selection. Overall appropriateness was 37.8%, compared with 72.1% for surgeons, although performance was substantially higher for monofocal IOL selection (21).

Together, these studies show a clear task gradient. Extraction of structured biometric measurements is relatively reliable, while performance becomes less consistent when models are required to integrate measurements into lens-power calculations, toric planning, or IOL selection. The main limitations are dependence on report layout, prompt design, and structured input, together with limited validation against postoperative outcomes.

### 3.2 Corneal topography/tomography and refractive surgery screening

Corneal imaging has been evaluated both for report interpretation and for refractive surgery screening. Tan and Coroneo assessed ChatGPT on 50 Pentacam reports and reported high accuracy in parameter extraction and diagnostic interpretation, although performance remained dependent on a controlled report format (14). This suggests that corneal tomography reports, which combine numerical indices with spatial maps, can be processed by general-purpose multimodal models, but robustness across devices, software versions, and display formats remains uncertain.

Choi et al. extended the task from interpretation to refractive surgery safety assessment in 200 eyes. ChatGPT integrated ocular measurements and corneal topography images, calculated ablation depth, residual stromal bed, percent tissue altered, and Ectasia Risk Score System values, and predicted surgical contraindications with an AUC of 0.977, sensitivity of 98.4%, and specificity of 97.1% (17). This study showed that general-purpose models can combine image interpretation with deterministic clinical calculations, although errors in formula selection or numerical reasoning remain possible when prompts are insufficiently constrained.

Overall, the evidence suggests that LLMs can support corneal report extraction, interpretation, and refractive surgery screening, but performance depends on input standardization and the degree to which calculations and decision rules are externally constrained.

### 3.3 Visual field and OCT-supported glaucoma decision support

Visual field interpretation represents another structured ophthalmic task in which LLMs have been evaluated. Tan et al. assessed ChatGPT on 60 visual field reports and reported complete extraction of global indices and reliability parameters, together with high diagnostic classification accuracy. Performance was weaker for more detailed defect characterization, with errors related to hemifield assignment and spatial localization (13). These findings suggest that extracting standardized visual field metrics is more reliable than interpreting complex defect patterns from plots.

Huang et al. extended this approach to multimodal glaucoma decision support by integrating visual field and OCT data from 204 eyes. ChatGPT achieved an AUC of 0.899, sensitivity of 96.0%, specificity of 83.7%, and overall accuracy of 85.2% against a consensus reference standard (16). This study moved beyond isolated report interpretation toward integration of structural and functional evidence for glaucoma assessment.

Together, these studies show that glaucoma-related applications span a progression from metric extraction to multimodal clinical inference (22, 23). Standardized parameters such as mean deviation, pattern standard deviation, and reliability indices are relatively suitable for structured extraction, whereas spatial defect interpretation and combined OCT–visual field reasoning require higher-level integration. The main limitations are small study numbers, limited external validation, and uncertainty about performance across different report formats, devices, and longitudinal disease stages.

### 3.4 Image-based interpretation across retinal, optic nerve, and external ocular imaging

Image-based studies extend the evidence beyond structured reports to direct visual interpretation of fundus photographs, OCT/OCTA, and external ocular images. Performance varies widely by modality and task (24, 25). In retinal imaging, ChatGPT and other MLLMs have been tested for diagnosis and grading of age-related macular degeneration, diabetic retinopathy, epiretinal membrane, and other retinal disorders (10, 26–33). Accuracy improves with few-shot prompting or added clinical context, but raw-image interpretation remains inconsistent, particularly for fine-grained staging and open-ended diagnosis (34, 35). Similar variability is seen in optic nerve assessment. Studies of glaucoma, optic disc swelling, and papilledema show moderate-to-high accuracy in some settings, but sensitivity, specificity, and agreement with experts differ substantially across datasets and prompts (22, 36–38).

External ocular and orbital imaging presents the same pattern. Models can estimate disease activity or support differential diagnosis, but performance improves when photographs are combined with structured clinical information (39, 40). Across these studies, image-only tasks are generally less reliable than multimodal inputs that include clinical context. The main limitations are dependence on dataset composition, image preprocessing, prompt design, and public case repositories, together with limited prospective validation.

### 3.5 Multimodal clinical reasoning and ophthalmic benchmarks

Multimodal studies evaluate whether general-purpose LLMs can integrate images, structured data, and clinical text for diagnosis, differential diagnosis, and management decisions. Across retinal, glaucoma, cataract, and mixed ophthalmic cases, performance often improves when clinically relevant context accompanies images, although the magnitude of benefit is model- and task-dependent (34, 41–44). Several studies using public case repositories showed substantially higher diagnostic accuracy with full clinical information than with image-only input, indicating that current models remain more reliable at integrating multimodal context than interpreting isolated ophthalmic images (45, 46).

Large benchmark studies further show that performance varies substantially across model families, subspecialties, and task types (47, 48). Ophthalmic visual question-answering and multimodal benchmarks have identified recurrent failure modes including misclassification (47), hallucination, unsupported reasoning, inconsistent responses, and failure to abstain. Educational and examination-based studies often report high overall accuracy, but these settings differ from real-world clinical workflows and may overestimate practical performance (49–53).

Overall, multimodal reasoning is one of the most promising but least standardized application areas. Its main strengths are flexible integration of heterogeneous inputs and broad task coverage. Its main limitations are dependence on clinical context, benchmark design, prompt structure, and possible exposure to publicly available cases. Future evaluation should prioritize prospective datasets, external validation, and clinically meaningful endpoints rather than benchmark accuracy alone (54).

## 4 Cross-cutting methodological limitations

Several methodological limitations recur across the current literature. First, study designs are highly heterogeneous. Sample sizes range from small single-center cohorts to large public benchmarks, and inputs include device reports, structured data, raw images, image-text cases, and educational question banks. Performance estimates therefore reflect different tasks and cannot be directly compared (47, 54). Second, many studies rely on public datasets or educational case repositories. These resources improve reproducibility but may not represent routine clinical workflows and may introduce data-contamination risk when publicly available cases overlap with model training data (55). External validation is uncommon, and most studies evaluate only one institution, one device format, or one benchmark dataset (56). Third, model performance is sensitive to prompting and input preparation. Repeated prompting, region-of-interest selection, few-shot examples, added clinical context, and image preprocessing can substantially change results. In several studies, performance changed substantially after additional prompting or changes in image framing, preprocessing, or clinical context (12, 34). Fourth, commercial LLMs are continuously updated. Studies performed at different times may evaluate technically different systems despite using the same model name, limiting reproducibility and longitudinal comparison. Reporting of model access date, interface, prompt, and inference settings is therefore essential (57, 58). Finally, many studies assess technical accuracy rather than clinical outcomes. High performance in extraction, classification, or formula replication does not establish safety in real-world care. Postoperative outcomes, prospective validation, calibration, failure detection, and clinician interaction remain limited (54). Future studies should therefore use standardized reporting, predefined prompts, independent external datasets, and task-specific clinical endpoints.

## 5 Safety requirements for clinical translation

Clinical translation requires safeguards matched to the task layer (59, 60). For information extraction, models should return structured outputs with predefined fields, explicit missing-value handling, and automatic checks for implausible ranges, laterality errors, unit mismatches, and cross-field inconsistencies. Report layout and image quality should also be verified before downstream use (59).

For interpretation and diagnosis, safety depends on traceable use of the available evidence. Models should identify the findings supporting their conclusions, distinguish observed features from inferred diagnoses, and avoid generating unsupported abnormalities. Performance should be validated across devices, institutions, disease spectra, and image-quality conditions. Because prompting and clinical context can strongly alter outputs, prompts and input formats should be standardized and documented (57).

For clinical decision support, additional safeguards are required because model errors may directly affect treatment or surgical planning. Deterministic calculations should be separated from free-form language generation and independently verified. Recommendations should be checked against predefined clinical rules and should include confidence or deferral mechanisms when inputs are incomplete, conflicting, or outside the validated scope (61, 62).

**Figure 2:**
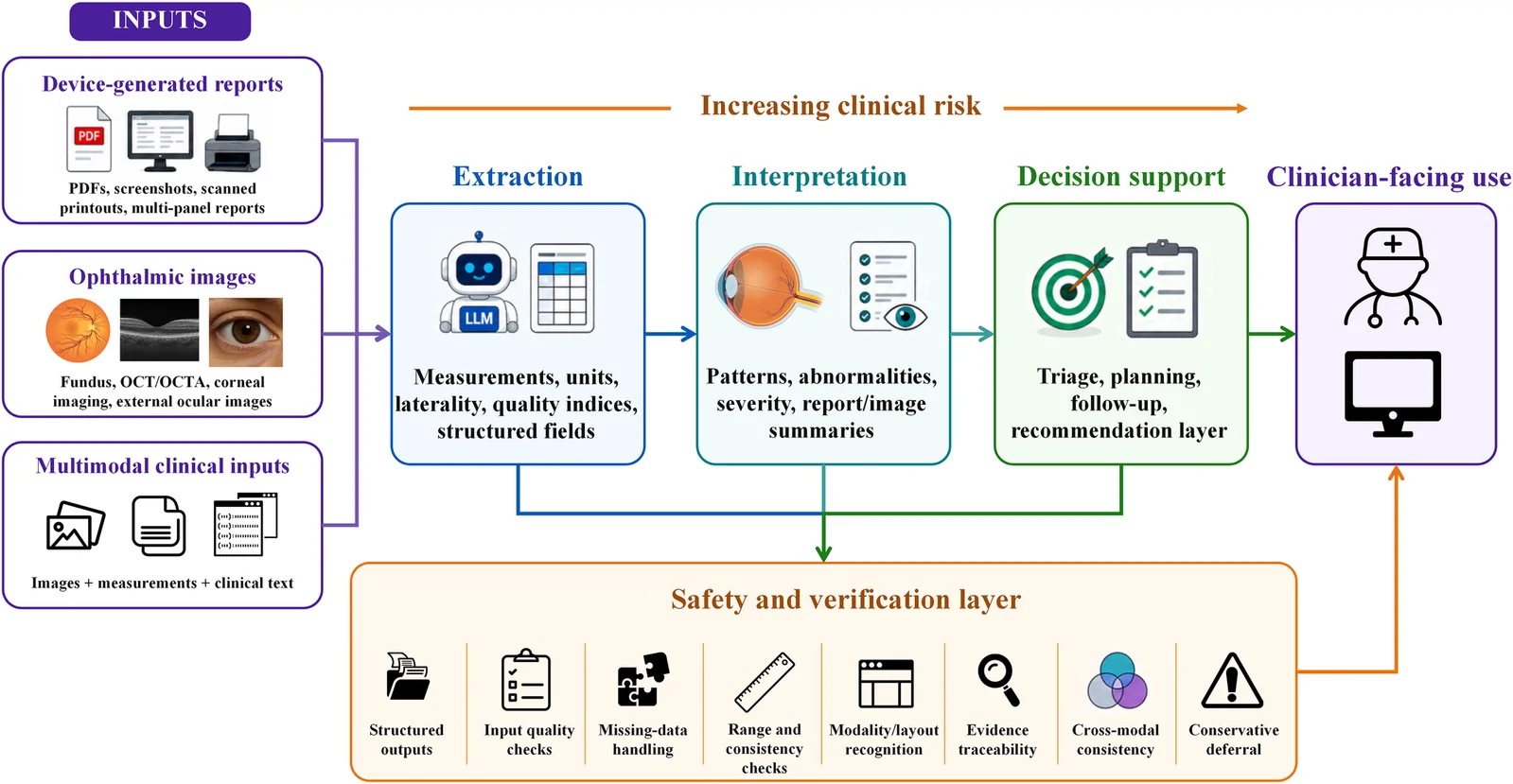
Task-layered framework for LLM-enabled ophthalmic examination understanding and clinical translation.

Human oversight remains necessary across all three layers. LLMs should function as assistive systems rather than autonomous decision makers, with clinicians retaining responsibility for verification and final decisions. Prospective studies should evaluate not only model accuracy, but also error detection, calibration, workflow integration, clinician override, and patient outcomes. A task-layered safety framework therefore provides a practical path from low-risk extraction toward higher-risk interpretation and decision support, with verification requirements increasing as clinical consequences become greater (Figure 2).

## 6 Discussion

The current literature shows that publicly available LLMs and MLLMs can process a wide range of ophthalmic inputs, but performance varies substantially with task complexity and input structure. The clearest pattern is a progression from relatively reliable extraction of standardized measurements to less stable interpretation of complex images and, finally, to the highest-risk use in clinical decision support. This task gradient is more informative than comparisons between model brands because the same model may perform well in constrained extraction yet poorly in open-ended diagnosis or treatment planning.

Input structure also has a major effect on performance. Models generally perform better when reports contain standardized numerical fields or when images are accompanied by structured clinical context. In contrast, raw image interpretation remains less consistent, particularly for spatial localization, subtle disease features, and complex multimodal integration. Several studies showed substantial gains after adding clinical information, using few-shot examples, selecting a region of interest, or constraining the prompt. These findings indicate that model performance reflects not only model capability, but also how the clinical task is framed and how information is presented (28, 36).

The main advantage of general-purpose models is their flexibility. A single publicly accessible model can potentially extract measurements, interpret reports, analyze images, reproduce calculations, and generate clinical recommendations without task-specific retraining. This differs from conventional ophthalmic AI systems designed for one disease or imaging modality (5). However, this flexibility also creates a reproducibility problem. Commercial models change over time, prompting strategies vary, and the same model name may represent different underlying systems across studies. Standardized reporting of model access date, interface, prompt, input format, and inference procedure should therefore become a minimum requirement for future studies (57).

The current evidence does not support direct comparison or pooling of performance across studies. Extraction accuracy from an optical biometry report is not equivalent to diagnostic accuracy from an OCT image or appropriateness of an IOL recommendation (63). Future evaluation should use task-specific endpoints and clinically meaningful reference standards. For extraction, relevant outcomes include field-level accuracy, laterality errors, and missing-value handling. For interpretation, studies should assess lesion recognition, diagnostic accuracy, calibration, and failure modes. Decision-support studies should prioritize prospective clinical validation, clinician override, and patient outcomes (56, 59).

This review also has limitations. The evidence base remains heterogeneous and includes retrospective cohorts, public image datasets, educational case repositories, and clinical decision-support studies. Some publicly available cases may overlap with model training data, and external validation remains uncommon. We therefore used qualitative evidence appraisal rather than formal pooling or a single risk-of-bias score.

Overall, publicly available LLMs and MLLMs are evolving from report parsing tools toward multimodal clinical assistants. Their most immediate role is in structured extraction and constrained interpretation, whereas diagnostic and decision-support applications require stronger validation and safeguards. Clinical translation should therefore follow a task-layered pathway in which verification, transparency, and human oversight increase with the clinical consequences of model error.
