# Supplementary material for: Large language models for ophthalmic examination understanding: from information extraction to clinical decision support
Source: Front Med (Lausanne). 2026 Sep 11;13:1937970. doi: 10.3389/fmed.2026.1937970 (PMC13612906; doi:10.3389/fmed.2026.1937970)
Supplement: Supplementary file 1 [file Table1.docx]

| **Supplementary Table S1 Database-specific search strategies used for study identification** | | |
| --- | --- | --- |
| **Database** | **Search strategy** | **Time range** |
| **PubMed** | ("large language model"[tiab] OR "multimodal large language model"[tiab] OR "vision-language model"[tiab] OR ChatGPT[tiab] OR "GPT-4"[tiab] OR "GPT-4o"[tiab] OR Gemini[tiab] OR Claude[tiab] OR DeepSeek[tiab] OR "generative AI"[tiab]) AND (ophthalm*[tiab] OR ocular[tiab] OR retina*[tiab] OR cornea*[tiab] OR glaucoma[tiab] OR cataract[tiab] OR keratoconus[tiab] OR "refractive surgery"[tiab]) | January 1, 2023–July 31, 2026 |
| **Web of Science Core Collection** | ("large language model" OR "multimodal large language model" OR "vision-language model" OR ChatGPT OR "GPT-4" OR "GPT-4o" OR Gemini OR Claude OR DeepSeek OR "generative AI") AND (ophthalm OR ocular OR retina OR cornea OR glaucoma OR cataract OR keratoconus OR "refractive surgery") | January 1, 2023–July 31, 2026 |
| **Scopus** | TITLE-ABS-KEY("large language model" OR "multimodal large language model" OR "vision-language model" OR ChatGPT OR "GPT-4" OR "GPT-4o" OR Gemini OR Claude OR DeepSeek OR "generative AI") AND TITLE-ABS-KEY(ophthalm* OR ocular OR retina* OR cornea* OR glaucoma OR cataract OR keratoconus OR "refractive surgery") | January 1, 2023–July 31, 2026 |
