# Supplementary material for: Large language models for ophthalmic examination understanding: from information extraction to clinical decision support
Source: Front Med (Lausanne). 2026 Sep 11;13:1937970. doi: 10.3389/fmed.2026.1937970 (PMC13612906; doi:10.3389/fmed.2026.1937970)
Supplement: Supplementary file 2 [file Table2.docx]

| **Supplementary Table 2 Included studies of LLM-enabled ophthalmic test-report understanding** | | | | | | | | | | | |
| --- | --- | --- | --- | --- | --- | --- | --- | --- | --- | --- | --- |
| **Study** | **Year** | **Report type** | **Sample size (n)** | **Input** | **Model(s)** | **Task layer(s)** | **Model access date** | **Interface** | **Inference settings** | **Key findings** | **Limitations** |
| Mihalache et al. ^[49]^ | 2024 | Multimodal retinal imaging | 69 reports | Image + Text | ChatGPT | Image interpretation; diagnosis | Dec 6–13, 2023 | ChatGPT Plus | No prior history | A definitive correct diagnosis was achieved in 50.7% of cases; the full patient description was required in 65.7% of correctly diagnosed cases, indicating strong dependence on textual context. | Educational retina-case dataset; diagnostic performance from images alone was limited; small case set and context-dependent results. |
| Mihalache et al. ^[48]^ | 2024 | Multimodal ophthalmic cases | 136 reports | Image + Text | ChatGPT | Image-based question answering; interpretation | Oct 16–23, 2023 | ChatGPT Plus | History cleared between cases | Across 429 questions from 136 cases, overall accuracy was 70%; performance was lower for image-based than nonimage-based questions (65% vs. 82%). | Educational multiple-choice dataset; performance varied by subspecialty and may not reflect open-ended real-world clinical interpretation. |
| Xu et al. ^[37]^ | 2024 | Multimodal ophthalmic imaging | 60 reports | Image | ChatGPT | Examination identification; lesion detection; diagnosis; decision support | Nov 14–26, 2023 | ChatGPT | History cleared; repeat test after 1 week | Across 600 responses, 30.6% were accurate, 21.5% highly usable, and 55.6% no harm; diagnosis accuracy was 16.1% and diagnostic repeatability was 63.3%. | Only 60 images across six modalities; open-ended prompting exposed major localization and lesion-recognition failures, and the model was not suitable for clinical decision-making. |
| Gill et al. ^[54]^ | 2024 | Oculoplastic image-based questions | 216 reports | Image + Text | ChatGPT | Education; image-based question answering | Mar-24 | ChatGPT | New chat/question | Image-based accuracy was 56.9%, significantly lower than text-only performance; some questions required an additional prompt to force image use. | Educational question-bank study; the model sometimes failed to use images, and source-question variability and stochastic outputs limit reproducibility. |
| AlRyalat et al. ^[24]^ | 2024 | Fundus photography | 400 eyes | Image | ChatGPT | Binary glaucoma classification | Nov 24–28, 2023 | ChatGPT | First response/image | Accuracy was 90% and specificity 94.4%, but sensitivity was only 50%; optic-disc cropping increased sensitivity to 87.5% while reducing specificity. | Single public benchmark; low sensitivity for unprocessed images and strong dependence on preprocessing; binary classification only. |
| Zhang et al. ^[25]^ | 2024 | Glaucoma multimodal clinical data | 26 reports | Image + Structured data | ChatGPT | Primary diagnosis; differential diagnosis | NR | NR | NR | Primary-diagnosis accuracy and completeness were lower than those of ophthalmologists, whereas differential-diagnosis accuracy was comparable and completeness was highest. | Only 26 convenience-sampled glaucoma cases; imaging was available only in some cases and subtype distribution was not prevalence representative. |
| Tan et al. ^[13]^ | 2025 | Optical biometry | 40 eyes | Image/PDF | ChatGPT | Extraction; decision support | NR | ChatGPT | NR | High extraction accuracy; IOL selection improved after region-of-interest specification. | Small cohort; single report family; prompt and ROI sensitive. |
| Jun et al. ^[21]^ | 2025 | Optical biometry | 180 eyes | Structured data | ChatGPT; Gemini | Formula replication; decision support | Aug–Sep 2025 | ChatGPT; Gemini | 2 runs/case | ChatGPT near-reference agreement with SRK/T; MAE 0.30 D; 78.8% within ±0.50 D. | Uses structured inputs rather than report images; formula replication, not postoperative refractive prediction. |
| Tan and Coroneo ^[15]^ | 2025 | Corneal topography/tomography | 50 eyes | Image/PDF | ChatGPT | Extraction; interpretation | NR | ChatGPT (Temporary Chat) | NR | High metric extraction and diagnostic agreement reported for corneal tomography interpretation. | Single controlled report family; robustness to layout/color scale/software drift uncertain. |
| Choi et al. ^[18]^ | 2025 | Corneal topography/tomography | 200 eyes | Image + Structured data | ChatGPT; Gemini; Llama | Calculation; contraindication prediction | NR | ChatGPT; Gemini Advanced; Hugging Face | New conversation/case | ChatGPT calculated RSB/PTA/ERSS and achieved AUC 0.977 for contraindication screening. | Retrospective; commercial-model dependence; formula hallucination possible without clear prompts. |
| Tan et al. ^[14]^ | 2025 | Visual field | 60 reports | PDF | ChatGPT | Extraction; interpretation | NR | ChatGPT | NR | 100% extraction of global and reliability metrics; high diagnostic classification; weaker defect-type classification. | Hemifield confusion and plot-localization errors clinically relevant; small sample. |
| Mikhail et al. ^[16]^ | 2025 | Multimodal clinical cases | 422 reports | Image + Text | ChatGPT | Diagnosis; next-step reasoning | NR | ChatGPT-4o | NR | With figures but no descriptions, diagnostic accuracy was 38.4% and next-step accuracy was 57.8%; adding figure descriptions restored diagnostic accuracy to 49.3%, near text-only performance. | Curated Clinical Challenges rather than real-time care; multimodal performance depended strongly on textual figure descriptions; model evaluated at a single time point. |
| Sorin et al. ^[43]^ | 2025 | External eye photography | 40 reports | Image + Text | ChatGPT | Image-text diagnosis | Nov 3 (year NR) | ChatGPT | New chat/query | Diagnostic accuracy increased from 47.5% with images alone to 67.5% with clinical context; adding context improved performance overall (p=0.033). | Small retrospective, deliberately selected case set; only external ocular photographs; comparator physicians were non-ophthalmologists. |
| Tomita et al. ^[45]^ | 2025 | Multimodal clinical questions | 580 reports | Image + Text | ChatGPT | Image-based clinical question answering; diagnosis | NR | ChatGPT | New chat/query | Multimodal ChatGPT achieved 77.1% accuracy, outperforming both an earlier multimodal ChatGPT configuration (71.0%) and text-only use (68.7%). | AAO question-based benchmark rather than real-world care; case-mix and model-version effects may limit generalizability. |
| Qin et al. ^[51]^ | 2025 | Multimodal ophthalmic imaging benchmark | 21,993 reports | Image + Structured data + Text | ChatGPT; LLaVA; InternVL; Qwen; VILA; Yi | Anatomical understanding; diagnosis; subgroup analysis | NR | GPT-4o; LLaVA; InternVL; Qwen; VILA; Yi | Default model-specific hyperparameters | Benchmarking 13 vision-language models revealed a marked performance drop in ophthalmology and six recurrent failure modes, including misclassification, failure to abstain, inconsistent reasoning, hallucination, unsupported assertions, and lack of domain knowledge. | Benchmark constructed from heterogeneous public datasets rather than prospective clinical workflows; performance varies substantially by task and modality. |
| Kiyat et al. ^[55]^ | 2025 | Image-based ophthalmology case questions | 174 reports | Image + Text | Claude | Image interpretation; education | NR | NR | NR | Accuracy was 89.7% for multiple-choice questions and 87.9% for open-ended questions, with no significant difference between formats and consistently high performance across subspecialties. | Educational platform rather than clinical workflow; one model was evaluated, and real-world diagnostic usefulness remains unvalidated. |
| Mehta et al. ^[44]^ | 2025 | Multimodal choroidal lesion imaging | 48 eyes | Image + Structured data | ChatGPT; Gemini; Perplexity | Diagnosis; treatment recommendation | Nov 1–Dec 1, 2024 | ChatGPT (Temporary Chat); Gemini 1.5 Pro; Perplexity Pro | History cleared/disabled between prompts | Gemini consistently outperformed ChatGPT and Perplexity; treatment-recommendation accuracy with clinical information was 72.5%, 64.7%, and 31.4%, respectively. | Small single-center retrospective cohort; limited to choroidal nevus and melanoma; performance remained below human graders and clinical context provided inconsistent benefit. |
| Gholami et al. ^[56]^ | 2025 | Ophthalmology examination questions | 3,357 reports | Image + Text | ChatGPT | Knowledge assessment; interpretation; decision-making | NR | Azure OpenAI Service | Independent API calls; 30 runs | The best ChatGPT reasoning configuration achieved about 91% overall accuracy and outperformed residents on recall, interpretation, and management questions; adding images improved model accuracy, although multimodal performance remained below residents. | Educational question-bank setting; multiple ChatGPT configurations and prompting strategies were used, limiting direct reproducibility and clinical generalizability. |
| Jalili et al. ^[40]^ | 2025 | Fundus photography | 300 reports | Image | ChatGPT | Glaucoma detection; feature identification | Dec 2023–Feb 2024 | OpenAI API | 2 runs/image | Glaucoma-detection accuracy ranged from 0.68 to 0.81 across three datasets; about 35% of images required repeated prompt submission, and agreement with experts varied widely. | Performance was dataset dependent; repeated prompting was often necessary, and optic-disc-centered preprocessing limits assessment of fully unprocessed clinical images. |
| Li et al. ^[38]^ | 2025 | Fundus photography | 1,074 reports | Image | ChatGPT; DeepSeek; Llama; LLaVA | Optic disc swelling classification | NR | Llama 3.2-vision; LLaVA; LLaVA-Med; GPT-4o; DeepSeek-VL2 | Model-specific default/recommended temperatures | Across 25 prompt-model pairs, accuracy ranged from 27.5% to 70.5%; the best single prompt-model pair used ChatGPT, while Llama showed the best average performance across prompts. | Large variability across models and prompts, frequent invalid/refusal responses, and no consistent benefit from increasing prompt complexity. |
| Shapiro et al. ^[39]^ | 2025 | Fundus photography | 198 reports | Image | ChatGPT | Papilledema detection | NR | ChatGPT | Separate chat/image | ChatGPT achieved about 86% accuracy with substantial agreement (κ=0.72), but specificity was only 78.9% and performance was inferior to the task-specific ResNet model and ophthalmologists. | Single public image dataset; relatively high false-positive rate and performance below dedicated image models and human experts. |
| Carlà et al. ^[31]^ | 2025 | OCT/OCTA | 50 reports | Image | ChatGPT; Gemini | Retinal image interpretation; diagnosis | 23-May-24 | ChatGPT-4o; Gemini Advanced | Conversation history retained | ChatGPT provided the correct diagnosis in 62% of cases versus 32% for Gemini and performed better in both surgical and medical retina cases. | Small exploratory dataset, single generic prompt, limited diagnostic spectrum, and model refusal or failure to recognize some imaging modalities. |
| Agbareia et al. ^[30]^ | 2025 | OCT | 772 reports | Image | ChatGPT; Claude | Retinal disease diagnosis | NR | OpenAI API; Anthropic API | 1 run/image/condition | Few-shot prompting improved ChatGPT accuracy from 56.3% to 73.1% and Claude from 40.0% to 71.0% across public OCT datasets. | Public datasets without clinical context; results were strongly prompt dependent and remained below established task-specific deep learning systems. |
| Most et al. ^[28]^ | 2025 | Fundus photography | 136 eyes | Image | ChatGPT; Gemini; Claude; Perplexity | AMD detection; severity grading; diagnosis | Dec 2024–Feb 2025 | ChatGPT; Claude; Gemini; Perplexity (paid) | Default (temperature 1.0); new chat/prompt | Compared four MLLMs for binary AMD classification, severity grading, and open-ended/multiple-choice diagnosis. | Single-center retrospective; model/task variability. |
| Most et al. ^[10]^ | 2025 | Fundus photography | 309 eyes | Image | ChatGPT; Gemini; Claude; Perplexity | DR detection; severity grading | Aug–Oct 2024 | ChatGPT; Claude; Gemini; Perplexity (paid) | New chat/image/prompt | Best binary DR accuracy modest; severity grading showed low sensitivity in source summary. | Performance fell short of clinical standards; strong model/prompt variability. |
| Chetla et al. ^[32]^ | 2025 | Fundus photography | 2,500 reports | Image | ChatGPT | Diabetic retinopathy grading | Jul 26–Oct 8, 2024 | ChatGPT | NR | Multiclass grading showed substantial bias toward no diabetic retinopathy; binary tasks performed better, but overall sensitivity and specificity for clinically relevant screening remained 47.7% and 73.8%. | Public dataset without clinical metadata; performance depended on prompt design and remained below established regulatory-approved diabetic-retinopathy AI systems. |
| Mihalache et al. ^[35]^ | 2025 | Fundus photography | 50 reports | Image | ChatGPT; Gemini; Copilot | Retinal disorder diagnosis | Mar 20–21, 2024; repeated weekly Jun 2024 | ChatGPT Plus; Gemini Advanced; Copilot Pro | 2 runs/image; history cleared between cases | No chatbot was sufficiently accurate for clinical diagnosis; accuracy improved markedly between March and June 2024, highlighting rapid performance change over time. | Only 50 fundus photographs, no clinical context, repeated testing across changing model versions, and substantial time-dependent model drift. |
| Kim et al. ^[41]^ | 2025 | External ocular photography | 165 reports | Image | ChatGPT; Gemini; Copilot; Clova X | Clinical Activity Score estimation; tool generation | NR | ChatGPT-4o; Gemini Advanced; Copilot; Clova X | Default; new session/evaluation | ChatGPT achieved mean absolute errors of 0.39 and 0.45 against ophthalmologist CAS ratings and AUCs of 0.974 and 0.990 for active thyroid-associated ophthalmopathy in two datasets. | Small public datasets, no patient metadata during image analysis, limited real-world validation, and repeatability concerns. |
| Lin et al. ^[20]^ | 2026 | Optical biometry | 54 eyes | Image/PDF | ChatGPT; DeepSeek | Extraction; toric IOL planning | NR | ChatGPT; DeepSeek | Default; new session/run; 3 runs/case | ChatGPT showed strongest agreement and most reliable axis-related extraction and planning. | Single-center retrospective workflow; calculator-based reference, not postoperative outcome validation. |
| Liu et al. ^[22]^ | 2026 | Cataract multimodal clinical data | 146 eyes | Image + Structured data + Text | ChatGPT | Clinical decision support | May–Aug 2025 | ChatGPT | New session/case; 5 runs/case | Overall IOL-selection appropriateness was 37.8% versus 72.1% for surgeons; performance was highest for monofocal IOL selection (90.0%), while agreement and consistency were poorer for more complex lens choices. | Single-center retrospective study with 74 patients; no postoperative outcome validation; low agreement with surgeons for complex IOL choices and potential sensitivity to model updates. |
| Huang et al. ^[17]^ | 2026 | Visual field and OCT | 204 eyes | Image/PDF + Structured data | ChatGPT | Decision support | Jan–Feb 2025 | ChatGPT o1 Pro | NR | AUC 0.899, sensitivity 96.0%, specificity 83.7%, accuracy 85.2% versus consensus diagnosis. | Higher-risk decision-support layer; needs calibration and longitudinal validation. |
| Lavista Ferres et al. ^[19]^ | 2026 | Structured clinical data | 1,419 reports | Structured data | ChatGPT | Data synthesis; diagnosis; assessment | NR | Azure OpenAI Service | NR | Overall accuracy was 0.76 and increased to 0.87 after excluding encounters with unsupported documentation or required ancillary tests; 6.6% of documentation-diagnosis mismatches were identified. | Single-center cross-sectional ED study; no imaging inputs; ophthalmologist-generated examination findings may overestimate performance in general ED settings. |
| Bulut and Reyhan ^[34]^ | 2026 | OCT | 90 reports | Image | ChatGPT | AMD feature recognition; diagnosis | Feb 20–25, 2025 | Julius AI | 2 runs/image | Agreement exceeded 90% for several OCT features, including image quality and fluid detection; sensitivity was lower for dry AMD (68.7%) and normal OCT (75.3%). | Small disease-specific dataset with only three groups; limited spectrum and platform/model-access dependence constrain generalizability. |
| Sato and Kuramoto ^[29]^ | 2026 | OCT | 250 eyes | Image | ChatGPT | ERM recognition; staging | May–Jun 2025 | ChatGPT | Memory disabled; 1 run/image | ChatGPT initially recognized ERM in 26.4% of cases; perfect agreement for Govetto staging was 46.0% with moderate weighted agreement (κ=0.513). | Single-center retrospective study focused only on idiopathic ERM; expert-discordant cases were excluded and initial disease recognition was limited. |
| Amiri et al. ^[46]^ | 2026 | Multimodal retinal cases | 40 reports | Image + Text | ChatGPT; Claude; Gemini | Diagnosis; differential diagnosis; treatment recommendation | Mar-25 | NR | 1 interaction/case; no carryover | With full context, ChatGPT had the highest diagnostic accuracy (78.4%); with image-only input, Claude performed best (73.7%). Gemini was substantially less accurate in both settings. | Small EyeRounds case set; curated educational cases rather than prospective clinical workflow; results are sensitive to context format and model updates. |
| Tabuse et al. ^[27]^ | 2026 | Fundus photography | 300 reports | Image + Structured data | ChatGPT; Gemini | Diabetic retinopathy triage; glaucoma referral | NR | ChatGPT; Gemini | Default; independent prompts; no memory | Performance was task dependent: ChatGPT showed moderate robustness for diabetic-retinopathy triage, whereas metadata could induce confirmation bias, particularly for Gemini; all tested models performed poorly for glaucoma referral. | Retrospective benchmark with structured prompts; metadata sensitivity and degenerate behavior in glaucoma referral limit clinical generalizability. |
| Jiao et al. ^[47]^ | 2026 | Multimodal ophthalmic cases | 261 reports | Image + Text | ChatGPT | Diagnosis; differential diagnosis; treatment recommendation | NR | ChatGPT | 1 run/case; no feedback | Diagnostic accuracy was 80.1% with full clinical context versus 54.7% with image-only input; signs/symptoms, differential diagnoses, treatment, and overall matching were all significantly better with full context. | Single-model study using public EyeRounds cases; full-context inputs included expert-generated image descriptions, and results may not represent raw-image-only clinical performance. |
| AAO = American Academy of Ophthalmology; AMD = age-related macular degeneration; AUC = area under the receiver operating characteristic curve; CAS = Clinical Activity Score; CLIP = Contrastive Language–Image Pretraining; D = diopter; DR = diabetic retinopathy; ED = emergency department; ERM = epiretinal membrane; ERSS = Ectasia Risk Score System; IOL = intraocular lens; LLM = large language model; MAE = mean absolute error; MLLM = multimodal large language model; OCT = optical coherence tomography; OCTA = optical coherence tomography angiography; OCR = optical character recognition; PDF = Portable Document Format; PTA = percent tissue altered; ROI = region of interest; RSB = residual stromal bed; SRK/T = Sanders–Retzlaff–Kraff/Theoretical; κ = kappa coefficient. | | | | | | | | | | | |
